# SME response to major exogenous shocks: The bright and dark sides of business model pivoting

**DOI:** 10.1177/0266242620936590

**Published:** 2020-08

**Authors:** Todd Morgan, Sergey Anokhin, Laurel Ofstein, Wesley Friske

**Affiliations:** Western Michigan University, USA; St. Cloud State University, USA; Western Michigan University, USA; Missouri State University, USA

**Keywords:** arbitrage, business model pivoting, exogenous shocks, innovation, opportunity selection

## Abstract

Within this short commentary, we explore the notion of pivoting; following major exogenous shocks, firms often contemplate business model pivoting where they change product or service offerings to capitalise on emerging opportunities. We assess the potential bright and dark sides of pivoting for new and existing firms in regard to quality of opportunities, fit with current capabilities and potential costs. The extant literature suggests that two forms of opportunities exist, arbitrage and innovation. We discern that post-shock, new firms may be better positioned to pursue arbitrage opportunities, whereas existing firms should target innovation. Existing firms may have more complications when pursuing arbitrage due to resource embeddedness and stakeholder obligations, and have a greater ability to innovate with an established resource base. Conversely, new firms can capitalise on arbitrage due to lack of embeddedness, as arbitrage requires a significant investment in opportunity selection. In addition, we offer suggestions for future research in regard to the current pandemic and more broadly, exogenous shocks.

Major exogenous shocks such as the COVID-19 pandemic unsettle the flow of economic processes and disrupt economic equilibrium (Li and Tallman, 2011). They also cause major distortions in labour markets and render – at least for a time – many prevalent business models ineffective. Although the existence of exogenous shocks is widely accepted by mainstream business scholars, their timing and severity cannot be forecast, and the extant literature overlooks how small business owners and entrepreneurs should respond to crises (Doern et al., 2019; Saridakis, 2012). Yet, relatively few studies examine how small firms survive during exogenous shocks or recover afterwards (Davidsson and Gordon, 2016; Devece et al., 2016; Pearce and Michael, 1997; Pearce and Robbins, 1994; Smallbone et al., 2012). When such attention is given, much of it focuses upon the social aspects of entrepreneurship in response to crises (Williams and Shepherd, 2016a, 2016b). Exogenous shocks put the very survival of small firms in doubt (Pal et al., 2014); unlike incumbents that may be ‘too big to fail’ and, as such, can negotiate special arrangements with policymakers to stay afloat due to their size and perceived importance to local or even national economies, smaller entrepreneurial ventures are often left with nebulous advice to ‘pivot’. Open questions remain as to whether such advice should be heeded and when pivoting is beneficial.

Entrepreneurship is generally seen as the individual-opportunity nexus (Shane and Venkataraman, 2000). Exogenous shocks have a major impact on the opportunities available to current and would-be entrepreneurs, and much like the nature of that impact differs substantially across industries and markets; entrepreneur responses also reflect these differences. Pivoting, defined as ‘a structured course correction designed to test a new fundamental hypothesis’ (Ries, 2011: 149), implies a purposeful search guided by evidence of a fundamentally better entrepreneurial opportunity that the firm in question is qualified to exploit. Therefore, not every action by a struggling entrepreneur would qualify as a meaningful pivot. The entrepreneurship literature distinguishes between necessity- and opportunity-driven entrepreneurial initiatives (Devece et al., 2016); while necessity motivates a substantial share of individual efforts at enterprising (Devece et al., 2016), it rarely results in purposeful testing of fundamental hypotheses in order to find the most attractive opportunity to pursue – an essential part of pivoting. Opportunity-driven initiatives appear more promising in this regard, although for *existing* small firms that promise is substantially constrained for a number of reasons.

Pivoting may be beneficial, and popular media abounds with examples of firms that have successfully repositioned themselves in response to exogenous shocks. Yet, not every change is a pivot, and those fundamental changes that do rise in response to pivoting come at a cost. The newly discovered opportunities may be of inferior quality for the firm in question, they may not be sustainable and the firm may be ill-positioned to take advantage of them. Pivoting itself may be a traumatic event in the life of the firm and like similar events – be that the initial founding of a new firm or the generational change in family firms – it could bring about business failure (Molly et al., 2010). Testing fundamentally different opportunities requires resources, which in the time of exogenous shocks may be severely constrained. Moreover, if such shocks reveal previously non-existent opportunities that become particularly prominent, one would expect a substantial growth of competitive pressure in that space, which will soon erode the above-average returns that the opportunity may provide. It thus appears that committing resources to pursue crisis-induced opportunities may be fraught with dangers such that a serious resource commitment may be risky. Only in those opportunities that do not necessitate a large strategic commitment of resources may digression be justified. The entrepreneurship literature identifies such opportunities as arbitrage opportunities, typically presented by the adage of ‘buying low and selling high’ (Kirzner, 1973). Even these opportunities, however, may become elusive as governments take control to attain stability when faced with shocks and may suppress opportunities to profit from price differentials. For instance, in the case of COVID-19, many governments restricted speculation in the face mask and sanitiser markets, thus denying entrepreneurs such opportunities.

It follows that despite all the opportunities they potentially create, exogenous shock periods may not necessarily be advantageous times for existing firms to pivot. In contrast, for *de novo* firms (i.e. new start-ups) the situation may be different. Due to labour market imbalance, a larger than usual share of qualified individuals may turn to the pursuit of such opportunities as an alternative to unemployment and given the lack of opportunity costs (which are indeed low for the unemployed), create new ventures to take advantage of the situation. Existing evidence suggests that firms created during major shocks tend to perform better over the long run (Cowling et al., 2015; Power and Reid, 2005; Smallbone et al., 2012). So, while existing small firms must exercise caution, new start-ups may benefit more from the disequilibrium introduced by exogenous shocks and establish a trajectory that will enable survival and success in the future.

## Exogenous shocks and their impact on opportunities

Exogenous shocks cause major disruptions to economic systems (Hudecheck et al., 2020). The COVID-19 pandemic, for instance, has generated disconnected supply chains, logistics challenges, shortage or unavailability of key resources, extreme price distortions, government restrictions on the functioning of many industries and markets, the need to redesign the working processes for many industries, consumer pessimism, and erosion of trust in global trade. For some industries, for example, healthcare-related or those shipping online orders may have profited from these disruptions (Valinsky, 2020). Conversely, the shock has caused major distress for many others; indeed, the difficulty of forecasting the duration of the crisis has motivated many existing firms to consider pivoting; changing their products, services, customers or markets. On the face of it, such changes may seem commendable with the media propagating the stories of successful pivots; for example, the exogenous shocks around 2008 when the housing bubble burst, in 2002–2003 with the SARS pandemic, and in 2001 as a result of the 9/11 terrorist attacks. More distant examples include the dot.com bubble burst, the HIV crisis, the polio epidemic of 1952, major wars and natural disasters. In many cases, existing firms have pivoted to capitalise on the changes introduced by the shocks; for instance, as a pivoting response to the SARS pandemic, Alibaba created the online shopping platform Taobao Marketplace to supplement its wholesale and B2B e-commerce sites, as millions of consumers turned to emerging online marketplaces to safely buy and sell goods amidst health concerns and government shutdown orders (Huddleston, 2020). The current COVID-19 pandemic has also seen a number of successful pivots such as Camp Gladiator, a fitness company created in 2008. Its original business model included bringing groups together for training sessions to run in public places to support one another through fitness. Following the pandemic, Camp Gladiator released its online option for virtual workouts, which allowed it to retain 97% of its customer base and acquire many new users. Compared with many traditional gyms that have been forced to close during generic shelter-in-place/stay at home orders, Camp Gladiator pivoted to online success (Ahuja, 2020).

These and many other examples seem to suggest that pivoting in times of exogenous shocks to take advantage of the newly created opportunities can be a winning strategy that should be encouraged. Indeed, information asymmetries created by the shock provide ample room for profiteering for an alert entrepreneur, and price disequilibrium along with supply and demand mismatch at levels unparalleled in recent history open opportunities for low-risk arbitrage opportunity pursuit. Moreover, the crises expose major gaps in available products and services and provide unique opportunities for innovators who can close the gaps and address the problems. The COVID-19 pandemic, for instance, has opened superior opportunities for firms developing ventilators, and the need for new vaccines virtually guarantees ample opportunities for firms that can develop and deliver them effectively and fast. Yet, small businesses need to exercise caution when considering pivoting options in response to the opportunities introduced by exogenous shocks.

## Are these opportunities worthy of a pivot?

Enterprising actions are said to occur in response to either necessity or opportunity, and many entrepreneurs cite crisis-related necessity in times of exogenous shocks as a reason to pivot (Devece et al., 2016). While this dichotomy appears to have face validity, in actuality one can never exploit necessity. Even ventures created from necessity pursue opportunities that may or may not be sufficiently attractive to warrant attention of other existing and would-be entrepreneurs. Accordingly, to understand whether pivoting is justified, one needs to consider the quality of opportunities created by exogenous shocks, their fit with the capabilities of firms in question and the cost of effectuating the pivot vis-a-vis staying the course.

Opportunities in the most general sense come in two basic forms – arbitrage and innovation (Anokhin et al., 2011). Arbitrage opportunities are concerned with information asymmetries and pricing disequilibriums, where the same resource may be priced differentially across markets (Kirzner, 1979, 1993). A particular case of arbitrage opportunities is technological arbitrage – where a new, more effective resource combination has been introduced to the market by some industry participant – but has not yet become widespread. This allows alert arbitrageurs, who typically operate in the same industry, to imitate the new technology and extract above-average returns until the market equilibrates to duly acknowledge the more effective way to combine resources (Anokhin et al., 2017). Opportunities of this nature motivate the entrepreneurial actions of new entrants (Anokhin and Wincent, 2014) and of existing firms (Anokhin, 2013) in excess of innovative opportunities. Exogenous shocks, such as that caused by the COVID-19 pandemic, introduce very pronounced opportunities for pure arbitrage – the proverbial ‘buy low and sell high’ (Kirzner, 1979) – and one may expect forward-thinking firms (Acar et al., 2010) and alert individuals to notice and attempt to exploit them.

However, arbitrage opportunities rarely justify a true business model pivot. Fundamentally new opportunities require substantial investments on the part of interested firms, and as such necessitate the presence of significant market demand over a period of time. Whereas new firms are still seeking opportunities and have not locked themselves into a particular trajectory, existing firms have a history that narrows their search options and may result in path dependency (Schreyögg and Sydow, 2011). Managers of established firms may develop a myopia that limits their ability to identify high-profile opportunities outside their field of expertise (Levinthal and March, 1993; Morgan et al., 2019; Shane and Venkataraman, 2000). As such, their ability to experiment is subject to core rigidities that effectively locks them into tightly constrained spaces (Leonard-Barton, 1995). Thus, attempts to explore loosely related opportunities brought about by exogenous shocks may take considerable time, effort, cost, result in inferior performance and so, may weaken the firm’s existing capabilities (Argyres et al., 2019). That is, when it comes to arbitrage opportunities, pivoting may be a better option for newly created firms whose capabilities have not yet ossified and who may turn their resources to the development of any competencies much like stem cells in biology. Business model pivoting includes rethinking one’s key partners, key resources, key activities, value proposition, customer relationships, channels, customer segments, cost structure and revenue streams (Christensen et al., 2016). For an established firm, the amount of ‘unlearning’ may be too burdensome to ensure success (Levinthal and March, 1993), particularly in light of the limited life expectancy of many arbitrage opportunities.

Innovative opportunities brought about by exogenous shocks, however, are a substantially promising direction for the pivot (Devece et al., 2016). By their very nature, such shocks are disruptive on many levels prompting both policymakers and consumers to realise the novel need as yet unaddressed by the existing businesses. For instance, in the case of COVID-19, policymakers are faced with the need to provide effective ventilators to hospitals to address the immediate pressures on the healthcare system and to introduce innovative vaccines as a means to minimise the outbreaks in the future. They also institute social distancing policies that necessitate the development of software to provide a wide spectrum of services from education to office meetings to R&D collaboration. Similarly, businesses reconsider how work is being done, which creates innovative opportunities for software developers and service providers. Unlike short-term price disequilibriums, these changes have a lasting impact on the society at large, which makes innovative opportunities stemming from exogenous shocks extremely attractive to existing firms. Moreover, inasmuch as firms scan the environment for such opportunities in close proximity to their competence base, they are advantageously positioned vis-a-vis *de novo* start-ups with respect to offering high-quality solutions that fit well with their history. That is, unlike arbitrage opportunities that are primarily attractive to newly created firms, innovative opportunities caused by exogenous shocks are particularly well suited for existing businesses. For this reason, small firms should focus on those as motivation for possible pivoting.

Given that entrepreneurship implies a nexus of individuals and opportunities, situations where one or both of these components are reconsidered may be highly traumatic for the firms. It is thus, hardly surprising that the new venture creation process where both elements of this dyad are new is fraught with the probability of failure such that a majority of new businesses do not survive beyond their first five years (Nobel, 2011; Wasserman, 2012). Similarly, family business generation transition is a highly traumatic event in the life of family firms (Molly et al., 2010), although just one part of this dyad – individuals in charge of the operations – is updated. In the same manner, one might expect pivoting to be linked to increased mortality of existing businesses because the other part of the dyad – opportunities targeted by the firm – is modified. It follows that existing firms would be wise to not rush into pivoting unless the opportunity in question is by demonstrably superior to that which they focused on previously and it fits well with their competence base. Conversely, new firms may have the luxury to pivot before they develop a distinct set of core competencies and so may pursue arbitrage opportunities that are generally more evident than innovative options (Anokhin et al., 2011). It is also important to note that firms created in the times of exogenous shocks tend to have higher long-term survival chances and, in many cases, exhibit higher performance (Terjesen et al., 2016). We suggest that this reflects the abundance of arbitrage opportunities available and the absence of core rigidities that may plague existing ventures.

## Beyond economics

Economic implications of pivoting notwithstanding, firms switching to address the urgent needs of the moment may tap into other pronounced benefits that make the choice of the appropriate pivoting strategy less well defined for existing small to medium-sized enterprises (SMEs). There is a sizable literature that explores social entrepreneurship initiatives rising in response to natural disasters (Chamlee-Wright and Storr, 2010; Dutta, 2017; Morrish and Jones, 2020). While there is still no universally agreed-upon definition of social entrepreneurship, most definitions include some focus on the simultaneous creation of social and economic value (Doherty et al., 2014; Saebi et al., 2019); it is generally understood that social venturing implies maximising value creation while satisfying value appropriation (Santos, 2012). Most for-profit businesses focus exclusively on the aspects of economic value; yet, in the long term the goodwill generated by working towards creating social value may translate into patronage and overall goodwill from the local community.

Adopting a social value approach may prompt existing ventures to consider pivoting, even in the absence of direct economic benefits to seek improved business–community relations. Extending a helping hand to the local community in times of crisis builds up social capital with current and future customers (Bin and Edwards, 2009). As an example of how small businesses may pivot to include a greater focus on social value, small, locally owned restaurants are donating meals to healthcare workers and other essential employees during the shelter-in-place order in certain states across the United States. While donations may not have been part of the restaurant’s business model, and it may be losing money with fewer customers, an investment in the business’s social value may lead to a new level of customer awareness and appreciation, and thus, greater patronage for the future. In this sense, expenses related to pivoting may be seen as investments in goodwill; discerning business owners may choose to sacrifice a little now for the prospect of future gains. In fact, one may think of this kind of pivoting as real options, where business owners keep future options open by making acceptable investments in projects that may not be immediately profitable to them.

While searching for ways to improve social value with customers strengthens many existing, and perhaps also attracts new customer relationships, the base expectations of these customer groups may shift during a market shock as well. Customer expectations around an SME’s ‘greenness’ or ‘sustainable business practices’ have increased (Bocken et al., 2014; DiPietro et al., 2013). For example, while customers may order take-out food from restaurants, they may choose to only patronise establishments which use biodegradable to-go containers. Similarly, while consumers may primarily order products from online retailers when local retailers are shuttered, they may pay a premium for products from firms or individuals using recycled materials, minimal packaging or a small batch production. Even retail giant Amazon.com has added a section for Small & Medium Businesses with sub-categories such as ‘Support Artisans’ and ‘Explore Family-Focused Businesses’ (see Helms and Dobson, 2016).

## Implications

Based on the analysis of opportunities offered by exogenous shocks, we offer some implications for decision makers at the firm level and for policymakers. Most importantly, firms should look at opportunity beyond necessity. Fortunately, many governments have offered a number of programmes to businesses such as forgivable loans that alleviate necessity to a substantial extent. Consequently, firms should consider the nature of opportunities they see emerging. Despite the few successful examples widely covered by the popular press, existing firms should be wary of shock-related pure arbitrage opportunities as they are unlikely to last sufficiently long and may require ‘unlearning’ on the part of the firms in question. Because of their learning trajectories, existing businesses may find it harder to exhibit adequate efficiency when pivoting away from their current products, markets, or both, as they lack the capabilities to simultaneously adjust the many elements of the business model (Morgan et al., 2019). At the same time, if their current capabilities could be applied to a generally novel innovative opportunity, this represents a promising basis for the pivot. While the number of such opportunities is limited, if one is found, pivoting is justified.

For policymakers, the important advice is to not fall prey to the public media obsession with pivoting. Rather, use the tools at their disposal to discourage existing businesses from unnecessary and potentially fatal pivoting while simultaneously easing business entry for new ventures with the potential to pivot more nimbly and effectively, and whose failure in doing so will not have a ripple effect of the same proportions. In that sense, the policies that allow existing businesses to survive such as forgivable loans, and for would-be entrepreneurs to try business venturing such as offering stimulus money, are well crafted and should be developed further. At the same time, policies aimed at suppressing the exploitation of pure arbitrage opportunities such as barring reselling face masks at a profit, while understandable politically and socially, are ill-conceived from the business point of view and can make the recovery harder due to eliminating the arbitrageur entrepreneur from the process. Effort should be afforded to communicating the temporary nature of many opportunities to those entrepreneurs considering pivoting with a public education campaign outlines the benefits and drawbacks of pursuing opportunities brought about by exogenous shocks.

## Future research directions

One unanswered question remains regarding why small firms pivot in the first place. Anecdotal evidence and examples of pivoting success stories in *Forbes* and *Inc.* suggest that most pivot from necessity in response to exogenous shocks. That is, the firm must change its business model in order to survive a crisis. However, entrepreneurship researchers have not actually determined whether most firms pivot from necessity, or pursue new opportunities arising from an exogenous shock or whether the pivoting decision is based on a combination of such factors.

Another set of unanswered research questions involves the entrepreneur’s approach to the arbitrage versus innovation decision. What factors affect the decision to pursue an arbitrage opportunity or to pursue innovation? Is a decision to pursue arbitrage during an exogenous shock simply a result of an entrepreneur’s low risk tolerance, cultural factors or other personal characteristics? And what role do societal institutions play in the decision? It may be easier for entrepreneurs in developing economies and economies in transition to take advantage of arbitrage opportunities during exogenous shocks if local markets are not as heavily regulated as developed countries and so, information asymmetries more pronounced. Moreover, developing economies and economies in transition may not have the societal resources, such as small business development centres and financing options (Kakapour et al., 2016), needed to develop innovations that have the most potential for long-term success. Furthermore, in an international context, entrepreneurs may be tempted by arbitrage when neighbouring countries have different regulations or policies that may be exploited, even if the returns are short-lived and the decision to pursue such opportunities is socially irresponsible.

There are a variety of other questions regarding the decision to pivot that remain unanswered. We have suggested that newer firms are relatively more likely to pivot than older ones as they are not restricted by resource lock-ins and path dependencies, but this relationship should be empirically examined. It also seems likely that entrepreneurs will explore ‘nearby’ pivots first before considering ‘distant’ pivots during crises. For instance, pivoting SMEs may leverage existing resources and capabilities to move into a related product category (e.g. local distilleries producing hand sanitizer during the COVID-19 pandemic) or modify distribution channels to reach customers in new ways (e.g. Camp Gladiator moving online during COVID-19 shelter-in-place orders). In contrast, some SMEs have successfully pivoted by seeking new resources and capabilities in order to produce completely unrelated products in new markets (e.g. Samsung was originally founded as a local, family owned grocery store, but it eventually grew and moved into textiles, petrochemicals, and electronics to take advantage of protectionist policies following the Korean War) (Bondarenko, 2020). Although both categories of pivots may result in success, more research is needed to determine whether one type of pivot tends to be more common and successful, on average, than others.

Finally, the entrepreneurship and crises literature maintains that entrepreneurs benefit emotionally from pivoting to social entrepreneurship in the face of a crisis (Williams and Shepherd, 2016b); we have noted how a pivot to social entrepreneurship may be a solid marketing strategy for weathering exogenous shocks. However, can the pivot to social entrepreneurship be a source of a long-term competitive advantage? Social entrepreneurship may result in positive publicity for the focal firm, higher levels of customer awareness, increased brand loyalty, and improved relationships with government regulators during and immediately following a crisis. But what happens to the SME when it returns to ‘business as usual’? Do those benefits disappear, or does the goodwill created by the social entrepreneur influence marketing success after the last effects of the exogenous shock disappear?

## Conclusion

In this note, we analysed the bright and dark sides of pivoting in response to exogenous shocks like the COVID-19 pandemic based on the notion of opportunity as agreed upon in the entrepreneurship literature. Rather than painting a broad-strokes picture, we present a more nuanced approach to explore pivoting, and suggest that arbitrage opportunities justify pivoting for new start-ups, whereas innovative opportunities should be in the centre of the pivoting agenda for existing firms. We also suggest that government policies have an important role in guiding the firms to successful pivots. Although much of our thinking was informed by the ongoing COVID-19 crisis, the applicability of our advice goes well beyond this particular event. It is our hope to encourage the dialogue along these lines with our fellow scholars, and our obligation to inform both policymakers and decision makers of the dangers inherent in trying to develop the one-size-fits-all approach to deal with exogenous shocks.
